# Sociocultural determinants of alcohol and cannabis use and misuse among Nunavimmiut

**DOI:** 10.17269/s41997-022-00733-6

**Published:** 2023-01-23

**Authors:** Yohann Courtemanche, Natalia Poliakova, Mylene Riva, Christopher Fletcher, Mireille Desrochers-Couture, Caroline Moisan, Camille Pépin, Sarah Fraser, Gina Muckle, Richard E. Bélanger

**Affiliations:** 1 Research Centre of CHU de Québec-Université Laval, Quebec City, Quebec Canada; 2 Canada Research Chair in Housing, Community and Health; Institute for Health and Social Policy and Department of Geography, McGill University, Montreal, Quebec Canada; 3 Department of Social and Preventive Medicine, Faculty of Medicine, Université Laval, Quebec City, Quebec Canada; 4 School of Psychology, Faculty of Social Sciences, Université Laval, Quebec City, Quebec Canada; 5 Faculty of Arts and Science, Université de Montréal, Montreal, Quebec Canada; 6 Department of Pediatrics, Faculty of Medicine, Université Laval, Quebec City, Quebec Canada

**Keywords:** Binge drinking, Cannabis, Inuit, Cultural characteristics, Social determinants of health, Hyperalcoolisation rapide, cannabis, Inuits, caractéristiques culturelles, déterminants sociaux de la santé

## Abstract

**Objective:**

Stemming from historical traumas and changes in the Inuit way of life, substance use and its intertwined problems are a major cause of concern for Nunavimmiut. This study’s objective is to investigate sociocultural determinants of substance use and misuse to inform culturally appropriate public health programs.

**Methods:**

The 2017 Qanuilirpitaa? survey was conducted among a sample intended to be representative of Nunavimmiut aged 16 and over (total *n* = 1326). Sociocultural factors included cultural identity, land-based activities, involvement in community activities, social support, and family and community cohesion. The frequency of binge drinking (5 or more drinks on one occasion), cannabis use, and problematic substance use (CAGE and DAST-10) were documented. Data were analyzed using weighted multivariate logistic regressions. Inuit partners were involved from the planning of analyses to the co-interpretation of results.

**Results:**

Nearly a third of Nunavimmiut aged 16 and over reported binge drinking at least once a week (29.3%), and 68.6% of drinkers were at risk of potential drinking problems. Forty-five percent (45%) reported using cannabis at least once a week, and 30% of drug users were at risk of potential drug abuse problems. Volunteering and participation in community activities were associated with lower odds of cannabis use, as was frequently going on the land with weekly binge drinking, potential drinking problems, and weekly cannabis use. Social support and community cohesion were associated with higher odds of weekly binge drinking, as was cultural identity (centrality scale) with potential drinking problems.

**Conclusion:**

Key determinants of substance use relevant to Inuit culture were identified. Results are in line with our Inuit partners’ experience in their communities and are coherent with current land-based interventions implemented in Nunavik. A thorough understanding of substance use contexts and related stressors should guide the content and implementation of substance use programs in Nunavik.

**Supplementary Information:**

The online version contains supplementary material available at 10.17269/s41997-022-00733-6.

## Résumé

**Objectif:**

Suite aux traumas historiques et changements au mode de vie, l’usage de substance et les problèmes associés sont une source d’inquiétude majeure pour les Nunavimmiut. L’objectif de cette étude est d’investiguer les déterminants socioculturels de l’usage de substances pour adapter les programmes de santé publique à la culture Inuit.

**Méthodes:**

L’enquête Qanuilirpitaa? 2017 repose sur un échantillon sélectionné pour être représentatif des Nunavimmiut (16 ans et plus; n = 1 326). Les déterminants socioculturels incluent : identité culturelle, activités sur le territoire, activités communautaires, support social, cohésion familiale et communautaire. La fréquence de consommation excessive d’alcool (5 consommations ou plus), l’usage du cannabis et l’usage problématique (CAGE et DAST-10) ont été documentés. Données analysées par régression multiple pondérée. Nos partenaires Inuit ont été impliqués de la planification des analyses à la co-interprétation des résultats.

**Résultats:**

Près du tiers des Nunavimmiut de 16 ans et plus ont rapporté une consommation hebdomadaire excessive d’alcool (29,3 %) et 68,6 % de ceux consommant de l’alcool étaient potentiellement à risque de consommation problématique. Quarante-cinq pourcent (45 %) ont rapporté consommer du cannabis au moins une fois par semaine et 30 % des consommateurs de drogue étaient à risque de consommation potentiellement problématique. Le bénévolat et la participation aux activités communautaires étaient associés à une cote plus faible d’usage de cannabis, tout comme la pratique fréquente d’activités sur le territoire avec la consommation hebdomadaire excessive d’alcool, d’usage potentiellement problématique d’alcool et d’usage hebdomadaire de cannabis. Le support social et la cohésion communautaire étaient associés à une cote plus élevée de consommation excessive hebdomadaire d’alcool, tout comme l’identité culturelle (centralité) avec l’usage potentiellement problématique d’alcool.

**Conclusion:**

Des déterminants clés de l’usage de substance spécifiques aux Inuit ont été identifiés. Les résultats concordent avec le vécu de nos partenaires dans leurs communautés et le cadre théorique d’interventions déjà implémentées impliquant des activités sur le territoire et de guérison. Les contextes de consommation et les stresseurs associés devraient guider l’élaboration des programmes au Nunavik.

## Introduction

The history of alcohol and drugs in Inuit communities is indissociable from colonization. The traumas caused by colonization—such as dog slaughters, residential schools, and diverse forms of abuse—were contemporarily accompanied by the introduction of alcohol in communities (Laneuville, 2015). Nowadays, substance use and its intertwined problems in communities is a social problem on its own. In the last three decades, alcohol and drug use in Nunavik have been increasing (Bélanger et al., 2020; Muckle et al., 2007). In 2017, 63% of Nunavimmiut over 16 years of age drank alcohol at least once a month and 29% binge drank (5 or more drinks on one occasion) at least once a week. Among alcohol users, 69% were at risk of problem drinking (Bélanger et al., 2020). Cannabis, the most widely used drug, was consumed by 63% of Nunavimmiut over 16 years of age, with 32% consuming cannabis daily in the preceding year. Furthermore, 32% of drug users were at risk of potential drug abuse problems (Bélanger et al., 2020). Substance use differed between sexes: while females were more likely to be at risk of potential drinking problems, men were more likely to use drugs.

Alcohol—and sometimes drug—abuse was identified as a major concern in a qualitative study conducted by Saturviit, the Inuit Women’s Association of Nunavik (Laneuville, 2015). Participants highlighted the consequences of substance use in their community, notably violence, crime, and the impact on the development of children. Substance abuse also impacts family and community life, as formulated by an elder: “Alcohol is the cause of lack of communication in the community. […] They are overtaken by alcohol, and nobody is working together” (Laneuville, 2015). Cultural identity has been hypothesized as a protective factor against substance use problems in Indigenous peoples, but studies conducted so far have contradictory results (Whitesell et al., 2012).

### Social determinants of Inuit health

Inuit Tapiriit Kanatami, a national organization for Canadian Inuit, has identified many social determinants that shape the health and well-being of Inuit. Social determinants of health, defined by the World Health Organization as “the conditions in which people are born, grow, work, live, and age, and the wider set of forces and systems shaping the conditions of daily life,” are universal factors, yet their manifestation in different communities is very much culture-specific (World Health Organization, 2022). In this paper, the Inuit-specific sociocultural factors studied were deemed relevant and further operationalized based on the participatory process between Nunavik representatives and researchers. Key determinants include the quality of youth development, culture and language, mental wellness, and the environment (Inuit Tapiriit Kanatami, 2014). Similar determinants were identified in the community component of the Qanuilirpitaa? 2017 Nunavik Inuit Health Survey (hereafter, Q2017 survey), a qualitative investigation of Inuit conceptions of health in Nunavik (Fletcher et al., 2021). In practice, these determinants take the form of three pillars identified by Inuit as central to their well-being: the family, talking/communication, and traditional Inuit cultural values and practices (Kral et al., 2011). Access to culturally sensitive health services is essential to promote and maintain health (Inuit Tapiriit Kanatami, 2014). The importance of Inuit culture is recognized and integrated in services and interventions, notably through the Inuit Values and Practices department created in 2007 by the Nunavik Regional Board of Health and Social Services (Nunavik Regional Board of Health and Social Services, 2021). Currently, timely access to addiction recovery resources in Nunavik remains difficult and often involves transfers to the south, complicating successful lasting changes and perhaps even being a deterrent to seeking care (Inuit Tapiriit Kanatami, 2014; Laneuville, 2015).

### Sociocultural factors and substance use

Quantitative research on the relationship between sociocultural factors and substances use among Inuit is scarce, with most studies relying on small samples and qualitative design (Laneuville, 2015; Robertson & Ljubicic, 2019). Various initiatives and activities grounded in these sociocultural determinants of health have been proposed and implemented in Nunavik communities (Makivik Corporation, 2019). Elders have suggested that teaching children and teenagers the old way of life on the land could prevent substance abuse (Laneuville, 2015). For example, Isuarsivik Regional Recovery Centre integrates land-based and cultural activities in their addiction treatment program. These activities include outings on the land, hunting, fishing, berry picking, sewing, beading, and carpentry (Isuarsivik Regional Recovery Centre, 2019). To our knowledge, there has been no quantitative evaluation of this intervention or any land-based recovery program.

In its 2020–2023 Strategy and Action Plan, Inuit Tapiriit Kanatami identifies as key objectives the advancement of Inuit-specific health policies, programs, and initiatives, based on Inuit-specific reliable data (Inuit Tapiriit Kanatami, 2020). In line with those objectives, this study will investigate sociocultural factors potentially related to substance use among Inuit of Nunavik, taking into account sociodemographic characteristics and differences between sexes. This study will focus on alcohol and cannabis use, the most prevalent psychoactive substances used in Nunavik (excluding tobacco), with concerning impacts on the communities.

## Methods

### Sample

This study used a probabilistic cross-sectional sample of 1326 Nunavimmiut who participated in the Q2017 survey. A complex multistage with replacement sampling plan was used to obtain a non-proportional stratified sample of the entire Nunavik population aged 16 and over. A list of all residents was obtained from the regional government prior to the survey, and was adjusted to take into account recent relocations (within and outside of Nunavik) as well as recent deaths. In addition to the objective of representativeness for the entire population, providing accurate estimations in subgroups of interest was a main goal of the survey. To achieve this, the Nunavik population was stratified according to sex, age (16–19, 20–30, and 31+ years old), and community (14 communities). Power analyses were conducted to evaluate the number of participants required in each stratum. The overall response rate was 31% for people aged 16 to 30 years old and 42% for people aged 31 years and over. This relatively low response rate was mainly due to non-contact rates and was taken into consideration in the computation of the sampling weights. Statisticians at the Institut national de santé publique du Québec computed sampling weights taking into account the sample design, non-response at recruitment, non-response on specific instruments, and adjustments to match sociodemographic characteristics of the Nunavik population (post-stratification).

### Community engagement and ethics

The Q2017 survey was set up following a resolution adopted by the Nunavik Regional Board of Health and Social Services (NRBHSS) requesting that a new health survey be conducted to update the information on the health status of Nunavimmiut. This survey was conducted in partnership with major Nunavik organizations, the Institut national de santé publique du Québec, and researchers from Université Laval, McGill University, and Trent University. An Inuit-led Steering Committee oversaw the preparation, conduct, data interpretation, and dissemination of the survey results in accordance with the First Nations principles of ownership, control, access and possession (OCAP®; First Nations Information Governance Centre, 2021). A Data Management Committee (DMC) evaluated the usefulness of the research questions for the region, and approved data and biological sample requests. This committee brings together representatives from the NRBHSS and the health centres, the Kativik Regional Government, the Makivik Corporation, the Kativik Ilisarniliriniq, the Avataq Cultural Institute, and the Qarjuit Youth Council. The proposal of this article was submitted to and evaluated by the DMC and community representatives in spring 2020 to ensure that the proposed analyses were within the scope of the survey’s original objectives. Thereafter, the first draft of this manuscript was presented to our Inuit collaborators and the results were discussed and co-interpreted in spring 2021. The final manuscript and a plain-language summary were shared with our partners. The Q2017 survey was also approved by the ethics committee of the Centre de recherche du CHU de Québec. Informed written consent was obtained from each participant, and a clinical follow-up for abnormal results was undertaken when needed. Detailed information on survey procedures is provided in the Methodological Report (Hamel et al., 2020).

### Measures

#### Sociocultural factors

Sociocultural factors identified by community members as important to Inuit wellness and health were investigated as potential determinants of substance use. These factors were grouped into four thematic blocks: (1) social support, (2) community activities, (3) traditional activities, and (4) cultural identity. A description of each measure can be found in Table 1. Detailed information on these factors has been published elsewhere (Muckle et al., 2020).

**Table 1:** Description of sociocultural indicators

Factor	Description
Block 1. Social support	
Affective social support	5 questions on frequency of three types of social support: positive interactions, emotional support, and love and affection (Richmond et al., 2007) Likert scale (1—all of the time to 5—never) Continuous score calculated by summing reversed scale (Cronbach *α* = 0.79)
Family cohesion	6 questions: 5 from the Brief Family Relationship Scale + one item to adapt to Inuit culture (Fok et al., 2014) Likert scale: 1—very true to 3—not true Continuous score calculated by summing reversed scale (Cronbach *α* = 0.78)
Community cohesion	4 questions on perception of social cohesion in the community Likert scale: 1—strongly agree to 5—strongly disagree Continuous score calculated by summing reversed scale (Cronbach *α* = 0.78)
Block 2. Community activities	
Participation in religious activities	1 question on participation in religious activities, services, or meetings (excluding weddings and funerals) Likert scale: 1—never to 4—one or few times a week Comparisons: participation at least once a month vs. less than once a month
Involvement in community activities	Frequency of involvement in two types of community activities: cultural/sporting community event or volunteering in a group or organization Likert scale: 1—always to 5—never Continuous score calculated by summing reversed scale (Cronbach *α* = 0.63)
Participation in healing and wellness activities	1 question on the participation in healing or wellness activities in past 12 months Yes/no answer
Block 3. Traditional activities	
Frequency of going on the land	From the spring until now, how often did you go on the land? Likert scale: 1—never, 2—occasionally, 3—often Comparisons: often, occasionally vs. never
Hunting and fishing	The highest frequency of hunting and fishing on four seasons during the previous 12 months Likert scale: 1—never, 2—less than once a month, 3—1 to 3 days per month, 4—once a week or more Comparisons: hunting or fishing at least weekly vs. less than weekly or never
Harvesting seafood	The highest frequency of harvesting seaweeds, mollusks, and urchins on four seasons in the previous 12 months Likert scale: 1—never, 2—less than once a month, 3—1 to 3 days per month, 4—once a week or more Comparisons: harvesting at least monthly vs. harvesting less than monthly or never
Berry picking	Frequency of seasonal berry picking in the previous 12 months Likert scale: 1—never, 2—less than once a month, 3—1 to 3 days per month, 4—once a week or more Comparisons: berry picking practiced at least monthly vs. practiced less than once a month
Abilities to practice traditional activities	4 questions on satisfaction with ability to go on the land, eat country food, and communicate in Inuktitut and satisfaction with cultural knowledge Likert scale: 1—very satisfied to 5—very dissatisfied Continuous score calculated by adding reversed scale (Cronbach *α* = 0.64)
Importance of spiritual values	“Do spiritual values play an important role in your life?” Yes/no answer
Block 4. Cultural identity	
Centrality	5 questions on importance of Inuit values from the Pacific Identity and Wellbeing Scale, adapted to Inuit culture Likert scale: 1—strongly agree to 5—strongly disagree Continuous score calculated by adding reversed scale (Cronbach *α* = 0.76)
Connectedness	6 questions on feeling of connection to Inuit and non-Inuit from the Pacific Identity and Wellbeing Scale, adapted to Inuit culture Likert scale: 1—strongly agree to 5—strongly disagree Continuous score calculated by adding reversed scale (Cronbach *α* = 0.65)

#### Substance use

The frequencies of alcohol consumption and of binge drinking in the previous year were self-reported. In this study, the focus is on binge drinking, which is a frequent mode of consumption that is of particular concern for Nunavimmiut, notably because of the consequences on families and communities (Laneuville, 2015). Binge drinking was defined for both males and females as having had five or more drinks on a single occasion (same evening, same party, etc.) (Naimi et al., 2003). A drink was defined as one bottle or can of beer, one glass of wine or wine cooler, one shooter, or one cocktail with 1.5 oz of liquor. The frequency of binge drinking episodes was rated on a 6-item scale from *never* to *more than once a week*. For analyses, frequency of binge drinking was dichotomized between weekly (once or more a week) and non-weekly binge drinking.

The CAGE questionnaire (Ewing, 1984) was used to assess potential problem drinking. This instrument is composed of four self-reported yes/no questions concerning alcohol use and its consequences. Two or more affirmative answers to CAGE questions are considered as an indication of problematic alcohol consumption with potential negative impacts on an individual’s daily life. The CAGE questionnaire was previously used in Nunavik’s 2004 Qanuippitaa? Survey, in Greenlandic Inuit, and has been validated in Native Americans (Bjerregaard et al., 2014; Muckle et al., 2007; Weatherall et al., 2020).

Cannabis use in the year preceding the survey, regardless of the type of products and modes of consumption, was also self-reported by questionnaire. Frequency was rated from *never* to *daily or almost daily*. For analyses, cannabis use was dichotomized as weekly (once a week or more) and non-weekly use.

Problems related to drug abuse were assessed by the DAST-10 screening tool. Derived from the DAST screening tool, the DAST-10 is a self-reported measure consisting of 10 yes/no questions concerning involvement with drugs, excluding alcohol and tobacco, in the past 12 months (Skinner, 1982). This screening tool covers all drugs; it is not a measure specific to cannabis use. In addition to contextual aspects of drug use (non-medical use of prescription drugs, more than one drug at a time), this screening tool also documents adverse physical (i.e., withdrawal symptoms, medical problems, blackouts) and psychosocial (i.e., family problems, illegal activities) consequences of drug use. The DAST-10 tool has been used and validated in different settings (hospital, primary care, self-assessment) and populations (Yudko et al., 2007). In Q2017, the DAST-10 had a moderate internal consistency (Cronbach *α* = 0.60), which is lower than what is usually reported (around 0.90) (Yudko et al., 2007). DAST-10 total scores were computed as the sum of affirmative answers (range: 0 to 10). For analyses, this score was dichotomized to represent potential drug abuse problems (moderate to severe level; score 3 to 10) versus no problems or low level (score of 0 to 2).

#### Sociodemographic characteristics

Several sociodemographic characteristics were considered as potential covariates: age, sex (males vs. females), marital status (single vs. married or common law vs. separated, divorced, or widowed), highest education level (from 1 = grade 1 to 15 = graduated from university), employment (employed/not employed), annual personal income (less than $20,000 vs. $20,000 and over), coastal region of residence (Hudson vs. Ungava), and community size (small vs. large) were documented by questionnaires.

### Analyses

Statistical analyses were conducted using SAS 9.4 (SAS Institute Inc., Cary, NC) for the entire population and for males and females separately. Analyses on potential drinking problems and drug use problems were conducted among users only (lifetime alcohol users and past-year drug users, respectively). Sampling weights were used for all estimates to be representative of the Nunavik population, and 500 bootstrap weights were used for variance estimations. The sampling weights were adjusted to account for non-response (Hamel et al., 2020). First, the distribution of sociodemographic characteristics, the prevalence of substance use, and potential problems were examined for all respondents, and then compared between sexes (using chi-square tests). Sociodemographic factors associated at *p* < 0.20 with the outcome in a first multivariate model (data not shown) were retained for all subsequent models. This liberal method of variable selection was used to ensure that all relevant factors were included in our analyses, as the potential bias resulting from underadjusting (residual confounding) is more concerning than the potential bias from overadjusting (unnecessary adjustment). To investigate sociocultural determinants, logistic regression models were first conducted in each of the four blocks of sociocultural factors (Table 3) to identify the best representatives of each thematic block (those associated with *p* < 0.10). Finally, regression analyses including all best representatives of each block of sociocultural factors in the previous step were estimated as a global model of determinants (between-block models; Table 4). Statistical significance was set at *p* < 0.05, and 95% confidence intervals were calculated.

## Results

Descriptive statistics on sociodemographic, sociocultural, and substance use variables are presented in Table 2. Significant differences between males and females were observed on most sociocultural variables: males reported higher levels of family cohesion, community cohesion, volunteering and participating in community activities, and frequencies of hunting/fishing and harvesting. Females reported higher levels of affective support, religious activities, healing and wellness activities, and frequencies of berry picking. Binge drinking was frequently depicted, with nearly a third of Nunavimmiut aged 16 and over reporting it at least once a week (29.3%). More than half of drinkers were at risk of potential drinking problems (68.6%). For cannabis, 31.6% reported using daily or near daily and 45.3% reported using at least once a week. Among drug users, 30.0% were at risk of potential drug abuse problems. Female drinkers had higher scores of problematic alcohol use. Females consumed cannabis less frequently, and female drug users had lower scores of drug-use problem compared to males.

**Table 2:** Descriptive statistics of sociodemographic, sociocultural, and substance use indicators in the Qanuilirpitaa? 2017 survey

Variable	Total (*n*_unweighted_ = 1326)	Females (*n*_unweighted_ = 873)	Males (*n*_unweighted_ = 453)	Comparison between sexes (*p* value)^a^
	Proportion (%) or mean			
Sociodemographic variables				
Sex	-	49.61	50.39	
Age (years)				
16–30	43.86	43.85	43.87	0.34
31–54	39.33	40.09	38.59	
55+	16.81	16.06	17.54	
Marital status				
Single	42.20	41.87	42.53	0.04
Married or common law	52.24	50.86	53.60	
Separated, widowed or divorced	5.56	7.26	3.87	
Education (years completed, mean)	9.16	9.34	8.98	0.01
Employment (% working)	68.25	67.09	69.40	0.41
Income				
<$20,000 per year	46.85	45.11	48.40	0.33
≥$20,000 per year	53.15	54.89	51.60	
Coast of residence				
Hudson Coast	56.63	56.27	56.97	
Ungava Coast	43.37	43.73	43.03	
Community size				
Small	42.66	41.91	43.39	0.04
Large	57.34	58.09	56.61	
Social support				
Affective social support (mean)	13.30	13.87	12.74	<0.001
Family cohesion (mean)	7.12	6.97	7.27	0.02
Community cohesion (mean)	11.56	11.17	11.95	<0.001
Participation in community activities				
Religious activities (monthly participation)	40.73	45.35	36.20	0.002
Volunteering and community activities	4.05	3.88	4.22	0.006
Participation in healing and wellness activities (% yes)	30.16	33.27	27.13	0.03
Traditional practices				
Frequency of going on the land				0.26
Never	13.41	12.34	14.46	
Occasionally	43.03	45.41	40.68	
Often	43.56	42.25	44.86	
Hunting/fishing (weekly)	62.60	58.29	68.86	<0.001
Harvesting (monthly)	35.41	32.59	38.21	0.03
Berry picking (monthly)	52.81	67.81	38.08	<0.0001
Ability to practice traditional activities (mean)	16.97	16.91	17.02	0.43
Importance of spiritual values (% yes)	82.63	72.76	80.32	0.05
Cultural identity				
Centrality (mean)	23.01	23.10	22.93	0.23
Connectedness (mean)	24.51	24.34	24.67	0.05
Substance use variables				
Frequency of binge drinking				
Never	27.10	30.09	15.29	0.09
Less than once a month	13.12	11.69	13.51	
Once a month	13.16	11.67	17.89	
2–3 times a month	17.27	16.65	14.62	
Once a week	12.50	11.48	14.53	
More than once a week	16.84	18.41	24.16	
CAGE total score^b^				
0	13.21	11.06	15.16	0.003
1	18.24	15.50	20.72	
2	24.91	23.31	26.36	
3	26.26	31.31	21.66	
4	17.37	18.80	16.09	
Frequency of cannabis use				
Never	36.61	46.95	26.35	<0.0001
Less than once a month	12.57	11.64	13.49	
1 to 8 times a month	5.52	4.94	6.10	
Once or twice a week	4.87	3.51	6.22	
3 to 4 times a week	8.86	7.20	10.52	
Daily or almost daily	31.56	25.76	37.32	
DAST total score^c^				
0	22.89	27.66	19.42	0.03
1	47.15	45.17	48.60	
2 or more	29.95	27.71	31.97	

^a^Weighted Wald log-linear *χ*^2^ test for proportions and *t* test for means

^b^CAGE questionnaire (Ewing, 1984), among those who drank alcohol in their life

^c^Drug Abuse Screening Test 10-items (Skinner, 1982), among those who used any drug in the past year

Results of within- and between-block models were similar; therefore, only between-block results will be presented in the text (within-block results can be seen in Table 3). Higher affective social support and community cohesion were associated with higher odds of weekly binge drinking (Table 4). Frequent activities on the land were associated with lower odds of weekly binge drinking and potential drinking problems among drinkers, but higher levels on the centrality scale of cultural identity were associated with higher odds for potential drinking problems. Lower odds of weekly cannabis use were observed for Nunavimmiut reporting more volunteering and community activities and those going on the land often (Table 4). Although not significant, the association between the frequency of land-based activities and potential drug use problems was similar as with other indicators (Table 4). Sex-specific models yielded similar results as analyses conducted on the entire sample. Although most are not significant, some associations appear to be stronger among males, some stronger among females. Notably, more frequent outings on the land were associated with lower odds ratios for weekly binge drinking and cannabis use among females than among males (Tables S1 and S2; Supplementary materials).

**Table 3:** Within-block multivariate regression analyses for substance use by sociocultural factors in the Qanuilirpitaa? 2017 survey

	Weekly binge drinking^a,b,c,d^ (*n*_unweighted_ = 1326)	Potential drinking problems^a,b,e^ (*n*_unweighted_ = 1246)	Weekly cannabis use^a,b,e^ (*n*_unweighted_ = 1326)	Potential drug abuse problems^a,c,d^ (*n*_unweighted_ = 797)
	AORs [95% CI]			
Block 1. Social support				
Affective social support^g^	1.04 [1.00, 1.09]	1.02 [0.98, 1.06]	1.00 [0.96, 1.04]	1.02 [0.96, 1.09]
Family cohesion^g^	0.95 [0.87, 1.02]	0.98 [ 0.91, 1.05]	0.92 [0.85, 1.00]	0.97 [0.88, 1.08]
Community cohesion^g^	1.08 [1.02, 1.15]	1.03 [0.97, 1.09]	1.03 [0.97, 1.10]	1.01 [0.92, 1.10]
Block 2. Participation in community activities				
Religious activities (ref = less than monthly)	1.09 [0.78, 1.52]	0.95 [0.70, 1.29]	1.04 [0.77, 1.40]	1.14 [0.74, 1.78]
Volunteering and community activities^g^	0.97 [0.90, 1.05]	1.00 [0.94, 1.07]	0.88 [0.81, 0.96]	0.98 [0.88, 1.10]
Healing and wellness activities (ref = no)	0.96 [0.69, 1.32]	1.00 [0.75, 1.34]	1.18 [0.86, 1.62]	1.21 [0.80, 2.07]
Block 3. Traditional practices				
Going on the land (ref = never)				
Occasionally	0.69 [0.43, 1.10]	0.70 [0.44, 1,12]	0.63 [0.38, 1.04]	0.81 [0.42, 1.54]
Often	0.56 [0.33, 0.95]	0.62 [0.38, 1.03]	0.42 [0.24, 0.74]	0.57 [0.27, 1.19]
Hunting/fishing (ref = less than weekly)	1.11 [0.75, 1.63]	1.06 [0.77, 1.45]	0.98 [0.66, 1.45]	1.18 [0.72, 1.94]
Harvesting (ref = less than monthly)	1.32 [0.93, 1.87]	1.24 [0.92, 1.68]	1.26 [0.88, 1.81]	1.00 [0.60, 1.67]
Berry picking (ref = less than monthly)	0.85 [0.61, 1.20]	0.86 [0.63, 1.17]	1.34 [0.94, 1.91]	1.19 [0.72, 1.93]
Ability to practice traditional activities^g^	1.02 [0.94, 1.10]	1.02 [0.95, 1.09]	0.98 [0.90, 1.05]	0.92 [0.83, 1.02]
Importance of spiritual values (ref = no)	1.18 [0.76, 1.83]	1.21 [0.84, 1.75]	1.12 [0.73, 1.72]	1.64 [0.58, 1.87]
Block 4. Cultural identity				
Centrality^g^	1.04 [0.96, 1.13]	1.10 [1.03, 1.19]	1.00 [0.92, 1.08]	0.98 [0.88, 1.10]
Connectedness^g^	1.03 [0.97, 1.08]	0.99 [0.94, 1.04]	1.00 [0.95, 1.06]	1.01 [0.93, 1.11]

*Note*: Logistic regression using sampling weights of a sample of 1326 participants for weekly binge drinking and cannabis use models. Potential drinking problems were evaluated among lifetime drinkers (*n* = 1246) and potential drug abuse problems among past-year drug users (*n* = 797)

^a^Adjusted for age, sex, and marital status

^b^Adjusted for employment

^c^Adjusted for region of residence

^d^Adjusted for community size

^e^Adjusted for education level

^f^Adjusted for income

^g^Continuous scores; see Table 1 for details on this measure. The odds ratio refers to an increase of one point on the scales

**Table 4:** Between-block multivariate regression analyses for substance use indicators by sociocultural factors in the Qanuilirpitaa? 2017 survey

	Weekly binge drinking^a,b,c,d^ (*n*_unweighted_ = 1326)	Potential drinking problems^a,b,e^ (*n*_unweighted_ = 1246)	Weekly cannabis use^a,b,e,f^ (*n*_unweighted_ = 1326)	Potential drug abuse problems^a,c,d^ (*n*_unweighted_ = 797)
	AORs [95% CI]			
Block 1. Social support				
Affective social support^g^	1.04 [1.00, 1.08]			
Family cohesion^g^			0.98 [0.91, 1.05]	
Community cohesion^g^	1.07 [1.01, 1.13]			
Block 2. Participation in community activities				
Volunteering and community activities^g^			0.91 [0.84, 0.99]	
Block 3. Traditional practices				
Going on the land (ref = never)				
Occasionally	0.69 [0.44, 1.09]	0.58 [0.35, 0,97]	0.72 [0.45, 1.14]	0.87 [0.47, 1.61]
Often	0.60 [0.38, 0.96]	0.59 [0.36, 0.98]	0.56 [0.35, 0.91]	0.70 [0.36, 1.38]
Ability to practice traditional activities^g^				0.95 [0.87, 1.04]
Block 4. Cultural identity				
Centrality^g^		1.10 [1.02, 1.18]		

*Note*: Logistic regression using sampling weights of a sample of 1326 participants for weekly binge drinking and cannabis use models. Potential drinking problems were evaluated among lifetime drinkers (*n* = 1246) and potential drug abuse problems among past-year drug users (*n* = 797)

^a^Adjusted for age, sex, and marital status

^b^Adjusted for employment

^c^Adjusted for region of residence

^d^Adjusted for community size

^e^Adjusted for education level

^f^Adjusted for income

^g^Continuous scores; see Table 1 for details on this measure. The odds ratio refers to an increase of one point on the scales

## Discussion

Our objective was to investigate sociocultural factors associated with substance use in a large-scale population survey. Many of our findings corroborate *Inuit Qaujimajatuqangit* (traditional knowledge), notably the link between the land, community activities, and well-being, as identified by Nunavimmiut in the survey’s community component (Fletcher et al., 2021). We found that frequent land-based activities and volunteering or participation in community activities were associated with lower likelihood of weekly binge drinking, potential drinking problems, and weekly cannabis use. These associations in a large population sample add to the growing literature suggesting a beneficial effect of the land on Inuit well-being (Robertson & Ljubicic, 2019), as put into practice in *by Inuit, for Inuit* interventions (Isuarsivik Regional Recovery Centre, 2019). The link between the land and well-being has been described in many communities across the Canadian North, notably by Nunavut Inuit who coined the phrase *Nunamii’luni quvianaqtuq*: *It is a happy moment to be on the land* (Robertson & Ljubicic, 2019). None of the land-based activities we investigated were individually associated with lower substance use, which may be due to the large spectrum of activities practiced by Nunavimmiut and local and regional specificities. The frequency of land-based activities, a more general indicator encompassing all types of activities, may be better suited to capture the diversity of activities.

Social support and family and community cohesion have been identified as protective factors against substance use in various populations (Cleveland et al., 2008; Hamme Peterson et al., 2010; Rapier et al., 2019). However, we observed that social support and community cohesion were positively associated with binge drinking. Our results may reflect the specificity of social networks and contextual characteristics of alcohol use in Nunavik communities. A study of social networks in Nunainguk (Labrador) reported overlapping contexts that provide social support and those linked to alcohol consumption (Moses et al., 2017). In this framework, associations between social support and alcohol use should be seen as co-occurring aspects in the social network rather than arising from any causal relationship (Moses et al., 2017). The analysis of prospective data would be required to clarify these relationships. Some measurement error may also arise from the interpretation of some statements used to compute the score (i.e., socially drinking with friends or going to a party may be linked to the item *someone to have a good time with* in the social support scale).

Over the last decades, alcohol and drug use has remained stable or increased in Nunavik, highlighting the need for interventions to reduce substance use and related harms. To be effective, such initiatives need to be supported by adequate long-term resources and local governance (Nunavik Regional Board of Health and Social Services, 2014). In a position statement by local stakeholders on the legalization of cannabis in Canada, Nunavik organizations highlighted that they do not currently have the capacity to provide sustained and culturally adapted educational campaigns or substance use recovery (Société Makivik; Conseil jeunesse Qarjuit; Régie régionale de la Santé et des Services sociaux du Nunavik and Kativik Ilisarniliriniq, 2017). Community-based activities and groups may be best able to provide help and support in a timely fashion, a factor identified by Nunavimmiut as a key to success (Laneuville, 2015). Support groups should also be culturally adapted; for example, while Alcoholics Anonymous meetings are readily available in most communities, other forms of group sessions such as healing circles may better suit participants (Fletcher & Denham, 2008).

Sociocultural factors that were not associated with substance use should not be ignored or deemed irrelevant to substance use, notably because of the difficulty to measure such complex, multidimensional concepts. While all measurement tools were chosen (and adapted as needed) in collaboration with Inuit representatives to ensure that they were culturally adapted, their performance has not been thoroughly studied in Inuit populations specifically. For example, accurately measuring episodes of heavy drinking (binge drinking) is particularly challenging given the episodic access to substances in Inuit communities. Therefore, our measure of binge drinking (5 drinks or more on a single occasion) may be subject to information bias, as pointed out in Korhonen (2004): “a person does not drink at all in a restricted or dry home community, but when s/he visits a community where there is a bar, s/he drinks to intoxication. Or a person does not drink during the week, but socializes on weekends by getting drunk.” In other words, binge drinking patterns should be understood considering the availability-scarcity context that is specific to these communities and may not be comparable to binge drinking in communities where alcohol is readily available. Additionally, all data collected were self-reported and some participants may have hidden or minimized their substance use (social desirability bias) which would bias our measures of association toward the null. Measures were put in place during data collection so that participants were confident in the confidentiality of their answers, notably by the completion of anonymized forms in private rooms.

Our investigation of sociocultural determinants of substance use was of an exploratory nature. Testing multiple hypotheses may result in some significant associations by chance. However, many associations were consistent across outcomes and the strength of those associations warrant attention regardless of significance. The similar associations reported between sociocultural factors and different outcomes may represent the associations between the outcomes themselves (i.e., the same participants may be at risk for alcohol use and cannabis use). Most indicators were documented retrospectively (lifetime, past year), but all were measured at the same moment. It is therefore impossible to draw conclusions on causality or even the directionality of associations. Reverse causation is also possible, as substance use may impact some factors measured. For example, substance users may have limited monetary resources because it is harder to hold a job or money is spent on alcohol or drugs, which are very expensive in Nunavik. Limited monetary resources may in turn limit their ability to acquire equipment necessary for on-land activities (Kishigami, 2000; Laneuville, 2015). Some associations reported, notably with the centrality scale of the cultural identity measure, are difficult to explain and may arise from uncontrolled confounding by factors not considered in our analyses. Cultural identity has been associated with negative mental health and substance use outcomes in other North American Indigenous groups, but the underlying mechanism remains unclear (Whitesell et al., 2012). When discussed with local representatives, measurement error has been raised as the most likely reason for such finding. Qualitative research could give us a better understanding of those results and better define a measure of cultural identity for Inuit. Finally, it is possible that some specific subgroups were less likely to be included in the survey’s sample, for example, heavy alcohol or drug users.

## Conclusion

Our investigation of sociocultural determinants of substance use in this large sample intended to be representative of Nunavimmiut allowed for the identification of several culturally specific factors. The results of this study support the theoretical framework used to develop many current initiatives to reduce substance use and its consequences by focusing on cultural and land-based activities and may guide future implementation.

## Contributions to knowledge

What does this study add to existing knowledge?

- Key determinants of substance use relevant to Inuit culture were identified, notably land-based activities.

What are the key implications for public health interventions, practice, or policy?

- These results support theoretical frameworks of interventions already implemented in Nunavik.
- Culturally relevant sociocultural determinants should be considered when crafting public health interventions.

### Supplementary Information

ESM 1 (DOCX 19 kb)

## Data Availability

Inquiries regarding access to the data should be addressed to the survey’s Data Management Committee.
