# Supplementary material for: Sociocultural determinants of alcohol and cannabis use and misuse among Nunavimmiut
Source: Can J Public Health. 2023 Jan 23;115(Suppl 1):114–25. doi: 10.17269/s41997-022-00733-6 (PMC10830961; doi:10.17269/s41997-022-00733-6)
Supplement: Supplementary file 1 — (DOCX 19 kb) [file 41997_2022_733_MOESM1_ESM.docx]

**Supplemental material**

**Table S1**

*Between-Block Multivariate Regression Analyses for Alcohol Use Indicators by Sociocultural Factors, by sex in the Qanuilirpitaa? 2017 survey.*

|  | **Weekly binge drinking ^a,b,c,d^** | | **Potential drinking problem ^a,b,e^** | |
| --- | --- | --- | --- | --- |
|  | **AORs [95% CI]** | | | |
|  | Females  **(n_unweighted_=873)** | Males  **(n_unweighted_=453)** | Females  **(n_unweighted_=817)** | Males  **(n_unweighted_=429)** |
| **Block 1. Social support** |  |  |  |  |
| Affective social support^f^ | 1.05 [1.00, 1.10] | 1.03 [0.95, 1.12] |  |  |
| Community cohesion^f^ | 1.01 [0.95, 1.07] | 1.18 [1.05, 1.33] |  |  |
| **Block 3. Traditional practices** |  |  |  |  |
| Going on the land (ref= Never) |  |  |  |  |
| Occasionally | 0.93 [0.53, 1.62] | 0.56 [0.26, 1.21] | 0.68 [0.37, 1.26] | 0.52 [0.24, 1.16] |
| Often | 0.70 [0.39, 1.24] | 0.54 [0.25, 1.17] | 0.55 [0.30, 1.00] | 0.66 [0.30, 1.45] |
| **Block 4. Cultural identity** |  |  |  |  |
| Centrality^f^ |  |  | 1.09 [0,99, 1.19] | 1.10 [0.98, 1.24] |

*Note*. Logistic regression using sampling weights of a sample of 873 females and 453 males for weekly binge drinking. Potential drinking problem was evaluated among lifetime drinkers (n=817 females, 429 males). ^a^ Adjusted for age, sex and marital status. ^b^ Adjusted for employment. ^c^ Adjusted for region of residence. ^d^ Adjusted for community size. ^e^ Adjusted for education level. ^f^ Continuous scores, see Table 1 for details on this measure. The odds ratio refers to an increase of one point on the scales.

**Table S2**

*Between-Block Multivariate Regression Analyses for Drug Use Indicators by Sociocultural Factors, by sex in the Qanuilirpitaa? 2017 survey.*

|  | **Weekly cannabis use ^a,b,e,f^** | | **Potential drug abuse problem ^a,c,d^** | |
| --- | --- | --- | --- | --- |
|  | **AORs [95% CI]** | | | |
|  | Females  **(n_unweighted_=873)** | Males  **(n_unweighted_=453)** | Females  **(n_unweighted_=469)** | Males  **(n_unweighted_=328)** |
| **Block 1. Social support** |  |  |  |  |
| Family cohesion^g^ | 0.97 [0.90, 1.05] | 0.99 [0.87, 1.12] |  |  |
| **Block 2. Participation in community activities** | |  |  |  |
| Volunteering and community activities^g^ | 0.87 [0.79, 0.96] | 0.93 [0.82, 1.06] |  |  |
| **Block 3. Traditional practices** |  |  |  |  |
| Going on the land (ref= Never) |  |  |  |  |
| Occasionally | 0.74 [0.42, 1.31] | 0.68 [0.31, 1.50] | 1.00 [0.50, 2.02] | 0.71 [0.25, 1.98] |
| Often | 0.48 [0.27, 0.88] | 0.62 [0.28, 1.38] | 0.78 [0.38, 1.57] | 0.86 [0.31, 2.39] |
| Ability to practice traditional activities^g^ |  |  | 0.97 [0.86, 1.09] | 0.88 [0.73, 1.05] |
| **Block 4. Cultural identity** |  |  |  |  |
| Centrality^g^ |  |  | 1.28 [0.65, 2.46] | 1.74 [0.85, 3.57] |

*Note*. Logistic regression using sampling weights of a sample of 873 females and 453 males for weekly cannabis use models. Potential drug abuse problem was evaluated among past-year drug users (n=469 females, 328 males). ^a^ Adjusted for age, sex and marital status. ^b^ Adjusted for employment. ^c^ Adjusted for region of residence. ^d^ Adjusted for community size. ^e^ Adjusted for education level. ^f^ Adjusted for income. ^g^ Continuous scores, see Table 1 for details on this measure. The odds ratio refers to an increase of one point on the scales.
